# Molecular Imprinting of Bisphenol A on Silica Skeleton and Gold Pinhole Surfaces in 2D Colloidal Inverse Opal through Thermal Graft Copolymerization

**DOI:** 10.3390/polym12091892

**Published:** 2020-08-22

**Authors:** Jin Chul Yang, Jinyoung Park

**Affiliations:** 1 School of Applied Chemical Engineering, Kyungpook National University, 80 Daehak-ro, Buk-gu, Daegu 41566, Korea; whdns861223@knu.ac.kr; 2 Department of Polymer Science & Engineering, Kyungpook National University, 80 Daehak-ro, Buk-gu, Daegu 41566, Korea

**Keywords:** colloidal lithography, silica inverse opal, gold pinholes, bisphenol-A, Freundlich isotherm

## Abstract

This study successfully fabricated BPA-imprinted poly(4-vinylpyridine-co-ethylene glycol dimethacrylate) (poly(4-VP-co-EGDMA)) quartz crystal microbalance (MIP-QCM) sensors on a silica skeleton surface and gold pinholes of silica inverse opal through surface-initiated atom transfer radical polymerization (SI-ATRP). The sensing features of the two MIP films on the structured silica surface and nano-scale local gold surface were investigated by measuring the resonant frequency change (∆*f*) in QCM sensors. The ∆*f* values for the *p*-MIP (MIP on gold pinholes) and *s*-MIP films (MIP on silica skeleton surface) were obtained with the ∆*f* value of −199 ± 4.9 Hz and −376 ± 19.1 Hz, respectively, whereas for *p*-/*s*-NIP films, the ∆*f* values were observed to be −115 ± 19.2 Hz and −174 ± 5.8 Hz by the influence of non-specific adsorption on the surface of the films. Additionally, the imprinting factor (IF) appeared to be 1.72 for *p*-MIP film and 2.15 for *s*-MIP film, and the limits of quantitation (LOQ) and detection (LOD) were 54.924 and 18.125 nM (*p*-MIP film) and 38.419 and 12.678 nM (*s*-MIP film), respectively. Using the Freundlich isotherm model, the binding affinity of the BPA-imprinted films was evaluated. This was measured in an aqueous solution of BPA whose concentration ranged between 45 and 225 nM. It was found that the *p*-MIP film (m = 0.39) was relatively more heterogeneous than the *s*-MIP film (m = 0.33), both of which were obtained from the slope of the linear regressions. Finally, the selectivity of the MIP-QCM sensors for BPA detection was determined by measuring the effect of other analogous chemicals, such as bisphenol F (BPF), bisphenol AP (BPAP), and bisphenol B (BPB), in aqueous solutions. The selectivity coefficients (k*) of the two MIP films had ~1.9 for the *p*-MIP and ~2.3 for the *s*-MIP films, respectively. The results reveal that, with respect to signal amplification of the QCM sensors, the s-MIP film has better sensing features and faster detection responses than the *p*-MIP film.

## 1. Introduction

Humans are exposed to high levels of toxic bisphenol A (2,2-bis-(4-hydroxyphenyl)propane; BPA) through dietary contamination, inhalation, and physical contact with BPA-containing materials [1]. This compound has widely been used in the plastic industry as an additive in the production of polycarbonate and epoxy resins. Food packing products, such as baby bottles, plastic bottles, and storage containers, are the end products [2]. Recent studies have focused on the possible etiologies of some human chronic diseases: diabetes, obesity, cardiovascular diseases, chronic respiratory, and kidney diseases [3]. Various sets of sensitive analytical equipment, such as gas chromatography-mass spectroscopy (GC-MS) and high-performance liquid chromatography (HPLC) have been extensively used to quantify the existence of BPA in humans, environmental resources, or industrial product samples. There are some limitations posed by the usage of these analytical tools, such as inconvenient operation and high cost. It is, therefore, necessary to develop simple and portable analytical systems capable of producing precise and accurate results.

The novel development of molecular recognition systems with artificial receptors using an imprinting technique has been highly interesting for effective separation systems and chemical/biosensors [4]. In the systems, a mixture of functional monomers, crosslinkers, and template target molecules in the presence of a porogen is essentially required. A molecularly imprinted polymer (MIP), which captures specific analytes in the selective functional cavities under the influence of covalent or non-covalent interactions, can be formed using thermal- or photo-polymerization. Studies have shown that various MIP structures, such as MIP spheres or nanoparticles with core-shell structures, improve the detection of BPA molecules [5,6,7]. Additionally, typical synthetic methods, such as surface-initiated atom transfer radical polymerization (SI-ATRP) [8] and electrochemical polymerization [9] have been implemented for the development of surface-imprinted films as BPA-detecting sensors. The SI-ATRP as one of the “grafting from” methods has extensively been used for surface modification on metals, SiO_2_, and polymers and has successfully controlled graft polymerization on the surface [10,11,12,13,14,15,16]. Following MIP synthetic strategies, the choice of efficient analytical equipment is necessary for the proper analysis of the sensing properties on MIP films based on confidential instrumentation. In recent studies, various analytical methods, such as optical [17], electrochemical [18], and gravimetric [19] analyses, have been used to detect trace amounts of BPA concerning improved sensitivity. However, quartz crystal microbalance (QCM), a piezoelectric sensor, offers simple operation and accurate detection by measuring resonant frequency changes (∆*f*) relating to the template mass loading (∆m). MIP film-modified QCM (MIP–QCM) sensors have been proven to be one of the most accurate and reliable analytical tools in use because numerous substances, such as viruses, proteins, and clinical biomarkers, including food and beverage applications, have been quantified using MIP-QCM [20].

In this study, BPA-imprinted poly(4-vinyl pyridine-co-ethylene glycol dimethacrylate) (poly(4-VP-co-EGDMA)) films were synthesized on a specific surface (silica skeleton and gold pinholes) of silica inverse opal-formed quartz crystals (QCs) using surface-initiated atom transfer radical polymerization (SI-ATRP). It was discovered that 4-VP functional monomers typically interacted with BPA in the MIP matrix through non-covalent hydrogen bonding, van der Waals force and π–π interaction [21]. Two MIP films, which were grown from silica skeleton or gold pinholes (*s*-MIP or *p*-MIP), were systematically designed to investigate the effects of the surface geometry and active surface area on which MIP films were thermally grafted on the sensing behaviors. Using the two different MIP films, the sensing effects of precisely designed MIP geometry on the structured silica surface and nano-scale local gold surface of QCM sensors, associated with analyte diffusion toward structured surfaces, were investigated. For that purpose, sensing properties, such as sensitivity and selectivity, were obtained by monitoring resonant frequency change (∆*f*) from QCM sensors, and the Freundlich isotherm model was applied in our MIP sensing systems and evaluated binding affinity.

## 2. Materials and Methods

### 2.1. Materials

Polystyrene (PS) latex beads (0.75 μm, 2.5% solid (W/V), which were purchased from Alfa Aesar Co. (Ward Hill, MA, USA), were served as a sacrificial monolayer mask for 2D silica inverse opal. Sodium dodecyl sulfate (SDS, Sigma-Aldrich Co., St. Louis, MO, USA) was used as an anionic surfactant for a PS bead monolayer, thereby facilitating buoyancy and highly packing at the air–water interface. Hydrochloric acid (HCl, Duksan Pure Chemicals Co., Ansan, Gyeonggi-do, Korea) and (3-mercaptopropyl)trimethoxysilane (3-MPTMS, Sigma-Aldrich Co., St. Louis, MO, USA) were used to chemically immobilize porous silica sol-gel films on 9 MHz gold-coated AT-cut Au-coated quartz crystals (QCs, QA-A9M AU[M], Seiko EG&G, Seiko instruments Inc., Chiba, Japan). Tetraethyl orthosilicate (TEOS, 98%, analytical reagent) and absolute ethanol (99.9%, analytical reagent) were supplied from Sigma-Aldrich Co., St. Louis, MO, USA. Trimethoxy(methyl)silane (TMMS) and cysteamine was purchased from Tokyo Chemical Industry Co., Tokyo, Japan. Triethylamine (TEA, Duksan Co.), 4-(dimethylamino) pyridine (DMAP, Sigma-Aldrich Co., St. Louis, MO, USA), 2-bromoisobutyryl bromide (2-BiBr, Alfa Aesar Co., Ward Hill, MA, USA), and anhydrous dichloromethane (DCM, 99.8%, Sigma-Aldrich Co.) were used to immobilize the initiator for atom transfer radical polymerization (ATRP). For MIP synthesis, 4-vinylpyridine (4-VP, Sigma-Aldrich Co., St. Louis, MO, USA), ethylene glycol dimethacrylate (EGDMA, Alfa Aesar Co., Ward Hill, MA, USA), bisphenol A (BPA, Sigma-Aldrich Co., St. Louis, MO, USA), and anhydrous acetonitrile (99.8%, Sigma-Aldrich Co., St. Louis, MO, USA) were, respectively, used as functional monomer, cross-linker, template molecule, and solvent. Copper (I) bromide (CuBr, Alfa Aesar Co., Ward Hill, MA, USA) was used as the catalyst and N, N, N′, N″, N″-pentamethyldiethylenetriamine (PMDETA, Sigma-Aldrich Co., St. Louis, MO, USA) was used as ligand. PMDETA was dried with calcium hydride (CaH2) which was obtained from Junsei Chemical Co. and distilled at low pressure. Bisphenol F (BPF), bisphenol AP (BPAP), and bisphenol B (BPB) were received from Sigma-Aldrich Co. Deionized (DI) water (Pure and Ultra-pure water system, pure ROUP30) and all other materials were used as received.

### 2.2. Preparation of the PS Colloidal Monolayer

A hexagonal close-packed colloidal monolayer was assembled as a sacrificial mask at the air–water interface and then transferred onto QCs after dropping an SDS aqueous solution [22]. First of all, glass substrates were ultrasonically treated in acetone, ethanol, and DI water for 5 min to remove impurities. The substrates were dried with gaseous nitrogen and further immersed in a piranha solution (98% H_2_SO_4_:30% H_2_O_2_ = 3:1 vol%) for 40 min to make the surface hydrophilic. Furthermore, the PS colloidal dispersion solution (80 μL) was then distributed on the pre-treated substrate using a micropipette and spin-cast at a spinning rate of 300 rpm for 5 min using a spin-coater (BGK, NSF-100DP). Lastly, the colloids were transferred to the water surface in a plastic Petri dish and the irregular polystyrene colloid monolayer was highly ordered by adding 2 wt% SDS solution (200 μL) in drops. The monolayer was placed on a QC substrate and completely dried for a day.

### 2.3. Preparation of the 2D Porous Silica Film

A silica sol-gel precursor solution was prepared following the previously reported procedure [23]. As the pre-hydrolyzation step, TEOS (5.32 mL), absolute ethanol (9.5 mL), and 0.1 N HCl aqueous solution (5 mL) were injected in a nitrogen-filled glass bottle and slightly stirred for 1 h. Absolute ethanol (17.83 mL) was added to the mixture and stirred for over an hour again. The solution was then processed through refrigeration before being used. The colloidal monolayer-coated QCs were immersed in a solution of 20 mM 3-MPTMS in ethanol for 2 h [24]. After washing with ethanol and drying with gaseous nitrogen, the 3-MPTMS-modified gold surface on the QCs was hydrolyzed and condensed in an aqueous solution of 0.1 M HCl for 2 h and then washed with DI water [25]. Furthermore, the pre-hydrolyzed silica precursor solution (10 μL) was dropped on the surface of the dried Si–O–Si network and spin-coated at 3000 rpm for 30 s. The coated substrate was then dried for 2 days in a desiccator at room temperature in order to form a robust silica network. Finally, the polystyrene colloidal monolayer on the substrate was thoroughly removed by immersion in toluene for 30 min.

### 2.4. Fabrication of the Molecularly Imprinted Polymers

Two independent active synthesis sites (gold pinhole and silica surface) on the 2D hexagonal porous silica films were prepared for grafting the BPA-imprinted films. To make the gold pinhole active for MIP synthesis, the silica skeleton on the QCs was blocked with a methyl group by soaking in a mixture of TMMS (28.8 mL), absolute ethanol (25 mL), and 0.1 N HCl aqueous solution (25 mL) for 4 h at room temperature. After rinsing with ethanol several times and drying with N_2_ gas, the QCs were immersed in an aqueous solution of 18 mM cysteamine for 4 h [26]. Subsequently, the immobilization of the initiator on the aminated pinhole which was placed in a nitrogen-filled glass bottle was performed by adding DCM (26 mL), TEA (0.05 mL), 2-BiBr (0.4 mL), and DMAP (0.04 g) to the bottle at 0 °C. The functionalization of the initiator was maintained with weak stirring at room temperature for 6 h [27]. After rinsing with DCM and drying with N_2_ gas, the MIP films were prepared as follows: the initiator-functionalized substrate was placed in an Erlenmeyer flask under N_2_ gas stream and a mixture solution containing 4-VP (0.42 mL, 4 mmol), EGDMA (5.68 mL, 30 mmol), and BPA (228 mg, 1 mmol) in anhydrous acetonitrile (25 mL) was injected into the flask, followed by the addition of the catalyst/ligand solution (14 mg CuBr and 32 μL PMDETA in 5 mL anhydrous acetonitrile). After purging with N_2_ gas for 10 min, the SI-ATRP reaction was carried out at 48 ± 2 °C for 24 h under gentle stirring. The MIP film was then rinsed with acetonitrile and dried with N_2_ gas to remove any remaining residues. Finally, the BPA molecules in the MIP film were extracted in methanol for 1 h. However, the methylation of silica surfaces and cysteamine immobilization were omitted for the *s*-MIP formation to make the silica surfaces active. The modification of the initiator and ATRP synthesis on the silica surfaces was followed by the aforementioned procedures. Finally, non-imprinted polymer (NIP) films were also fabricated using the same procedure but in the absence of BPA molecules.

### 2.5. Adsorption Behaviors of MIP-QCM Sensors

MIP/NIP film sensitivity was evaluated by monitoring in a 125 mL aqueous solution with BPA concentrations ranging from 45 to 225 nM for a 1-h rebinding process. Furthermore, the sensing response was recorded in the respective aqueous solutions containing 225 nM BPA and its analogs (i.e., BPF, BPAP, or BPB) to evaluate the selectivity of the QCM sensors for recognizable BPA binding sites. With the use of the Sauerbrey Equation, the average resonant frequency change (∆*f*) obtained was converted to the mass of adsorbed analyte [28]. For 9 MHz AT-cut Au-coated QCs (sensitivity factor ≈ 0.1834 Hz cm^2^ ng^−1^), a decrease of 1 Hz in the ∆*f* value was almost equal to 1.07 ng in the amount of mass loaded on the total gold area (A = 0.19625 cm^2^) [29]. The Δ*f* values of the MIP and NIP films were measured in the air before and after the BPA-extraction process using a QCA 922 quartz crystal analyzer (Seiko EG&G Co. Ltd., Tokyo, Japan) to determine the mass of the BPA-free polymer matrix and calculate the maximum binding capacity (Q_max_).

### 2.6. Characterization of Structured MIP Films

Atomic force microscopy (AFM, NX20, Park Systems, Suwon, South Korea) and field-emission scanning electron microscopy (FE–SEM, Hitachi SU8220, Tokyo, Japan) were used to analyze the surface topography of the MIP films on porous silica sol-gel structures.

## 3. Results and Discussion

### 3.1. MIP/NIP Imprinting on the Structured Surface

**Figure 1:**
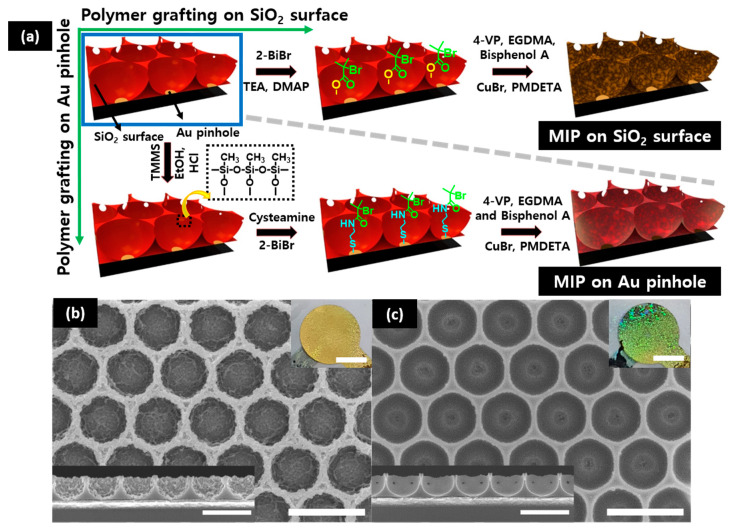
(**a**) The fabrication process of the *s*-MIP film (grown from the only silica surface) and *p*-MIP films (formed on the gold pinholes) on 2D porous silica thin films and (**b**,**c**) scanning electron microscopic (SEM) images of (**b**) *s*-MIP film and (**c**) *p*-MIP films. The insets represent the side views of the two MIP films (scale bars are 1 μm) and the photographs of MIP-based QCM sensors (scale bars are 0.25 cm).

A hexagonally packed PS colloidal monolayer was used as a sacrificial mask in which the silica precursor was filled into the voids of PS colloids arranged on the QCs. A well-ordered porous structure with a gold pinhole at the center of each pore was obtained after the formation of the silica sol-gel network and the removal of the PS colloid by toluene rinsing (Figure S1). As shown in Figure 1a, a surface initiator was used to modify either the silica sol-gel skeleton or the gold pinhole for further synthesis steps. To systematically fabricate poly(4–VP–co–EGDMA) MIP (or NIP) films on the QCs, SI-ATRP synthesis was performed on the surface of the structured silica and the gold pinhole. The SEM images, as shown in Figure 1b,c, reveal that the BPA-imprinted MIP films were successively formed at different local areas—i.e., on the silica (*s*-MIP) or the gold pinhole (*p*-MIP). For more clarity of the MIP formation, AFM images are shown in Figure S2. BPA-imprinted polymer growth was observed in the silica skeleton for the *s*-MIP film. The *s*-MIP film grew on the entire surface of the silica skeleton and exhibited a considerably rough surface, whereas the *p*-MIP film formed only near the gold pinhole area. In both MIP films, structurally recognizable functional cavities were easily generated by breaking the non-covalent bond between the BPA templates and the functional monomers in the polymer matrix. In general, structured MIP-QCM sensors could show amplified ∆*f* signals in a limited sensing period compared with the simple planar films because of an increased surface area to volume (S/V) ratio [30]. The thickness of the MIP films could also influence the sensing response and period of the structured MIP films, which affects controlled diffusion behaviors in a polymer matrix. Therefore, the precise control of these synthetic routes for MIP growth on specific surface areas should be considered for the development of MIP films with a fast sensing response and a high sensitivity.

### 3.2. BPA Sensing Response

**Figure 2:**
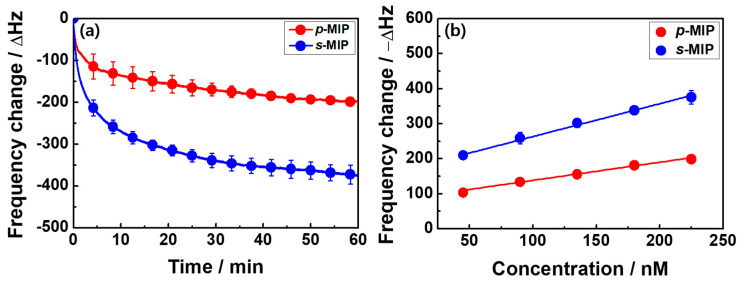
(**a**) Frequency change (∆*f*) as a function of time on the surface of two MIP films (*p*-MIP and *s*-MIP) in a 225 nM BPA aqueous solution for a 1-h rebinding process (n = 3) and (**b**) −∆*f* values on the *p*-MIP and *s*-MIP films as a function of the initial BPA concentration (C_0_, 45–225 nM) for a 1-h rebinding process.

The ∆*f* value, depending on BPA binding to accessible cavities for the two MIP films (i.e., *p*-MIP and *s*-MIP films), was monitored in a 225 nM aqueous solution (125 mL) of BPA for an hour rebinding process. As shown in Figure 2a, the ∆*f* values for both imprinted films increased gradually with rebinding time, indicating that the BPA molecules were predominantly diffused into the films and non-covalently bonded to functional nanocavities. The sensing signal of the *s*-MIP film was amplified (∆*f* value of −376 ± 19.1 Hz) compared with the ∆*f* value for the *p*-MIP film (−199 ± 4.9 Hz). When the ∆*f* value of each film was converted into the appropriate Q_e_ values (i.e., the equilibrium binding capacities, ∆m_BPA_/∆m_BPA-free polymer_), the Q_e_ values could be comparatively evaluated, resulting in a value of 134 ± 3.3 mg/g for the *p*-MIP film and 73 ± 3.7 mg/g for the *s*-MIP film (Figure S3). The maximum binding capacity (Q_max_) of both MIP films was calculated by mass difference before and after BPA removal from the MIP films. Although the MIP mass grown on the silica surface was much larger than that on the gold pinhole, the *p*-MIP film with Q_max_ (170 mg/g) film had more recognizable cavities than the *s*-MIP film (Q_max_ = 115.4 mg/g), owing to the effects of film growth and thickness. According to the Q_max_ and Q_e_ values, 78.7% of the binding sites in the polymer matrix were filled with BPA molecules on the *p*-MIP film, whereas the corresponding value for the *s*-MIP film was 63.7% for a 1-h rebinding process because of the thicker MIP film forming on the silica surface. For *p*-/*s*-NIP films, the ∆*f* values were obtained in a relatively lower range between −115 ± 19.2 Hz and −174 ± 5.8 Hz (n = 3), owing to the influence of non-specific adsorption on the surface of the NIP films in the absence of functional cavities (Figure S4). The ∆*f* values for this non-specific adsorption could depend on the surface area of the NIP. Based on the ∆*f* value obtained, the imprinting factor (IF)—i.e., ∆*f*_MIP_/∆*f*_NIP_ [31]—was 1.72 and 2.15 for the *p*-MIP and *s*-MIP films, respectively.

### 3.3. BPA Binding Affinity Assessment

The BPA binding on the two different MIP films was measured using the ∆*f* values in aqueous solutions of BPA with the concentration ranging between 45 and 225 nM (Figure S5). As shown in Figure 2b, the binding behaviors of all MIP films exhibited a linear increment in the ∆*f* values with increasing BPA concentrations. The increased −∆*f* value of the *p*-MIP film indicated a linear regression Equation of −∆*f* = 0.518 C_0_ ± 86.61 with a correlation coefficient of 0.992. However, the sensing signal of the *s*-MIP film had a value of 0.939 C_0_ ± 169.57 and its accompanying correlation coefficient was 0.998 in the examined concentration range. Furthermore, based on the Equations LOQ = 10 (*S/m*) and LOD = 3.3 (*S/m*), where S is the standard deviation of the intercept and m is the slope of the regression line, the limits of quantitation (LOQ) and detection (LOD) were calculated from linear calibration curves for the two MIP films in Figure S6, which revealed a change in the binding capacity as a function of BPA concentration [32,33]. The LOQ and LOD values were 54.924 and 18.125 nM (*p*-MIP film) and 38.419 and 12.678 nM (*s*-MIP film), respectively. These results indicate that the *s*-MIP film maintained relatively lower quantitative limits than the *p*-MIP film. Similarly, the two NIP films were also investigated, as shown in Figures S7 and S8.

**Figure 3:**
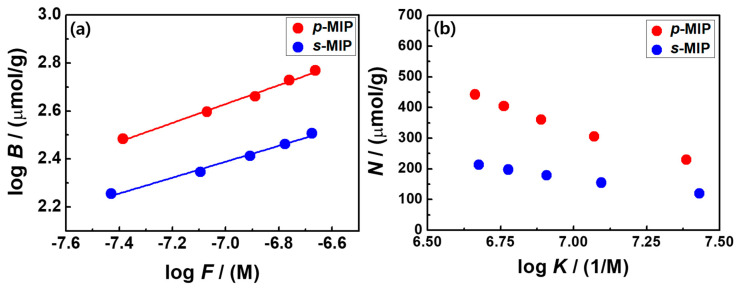
(**a**) Plot of log *B* versus log *F*, where *B* is equal to the Q_e_ values (μmol/g), and *F* is the concentration of the free analyte in solution after 1-h BPA adsorption; (**b**) affinity distributions of *p*-MIP and *s*-MIP films derived from the Freundlich model plotted against N formats as a function of log *K*, where *K* is the affinity constant in 1/M and *N*(*K*) (μmol/g) is the number of binding affinities for a given affinity.

For the BPA-imprinted QCM sensors, the Freundlich isotherm model was applied to assess the binding behaviors on the heterogeneous MIP films within the examined concentration range [34,35]. The micromoles of the bonded BPA per unit gram of poly(4-VP-co-EGDMA) (μmol/g) (*B*) were recalculated from the sensing response (∆*f*) of the MIP-QCM sensors, and the concentration of the free analyte (*F*) in the remaining solution after a 1-h rebinding process was gained from the initial concentration (C_0_) and the bonded BPA mass. As shown in Figure 3a, the log–log format of the model shows the linear regression for BPA adsorption of both the MIP films. The fitting parameters (i.e., *m* and *a*) of the Freundlich model, as shown in Equation (1), indicate the Freundlich heterogeneity index and the pre-exponential factor including the Freundlich capacity (*N*_T_) and average affinity (*K*_0_) parameters, respectively.

log *B* = *m* log *F* + log *a* (1)

The *p*-MIP film (*m* = 0.39) was relatively more heterogeneous than the *s*-MIP film (*m* = 0.33), which was obtained from the slope of the linear regressions. Approximations of the exponentially decaying distribution, in association with the continuous affinity distribution expression for the *N* and *K* values, could be predicted by the Freundlich model using the Equation (2).

*N*(*K*) = 2.303*am*(1 − *m*^2^)*e*^−2.303*m* log*K*^ (2)

where *K* is the affinity constant in 1/M and *N*(*K*) is the number of binding sites for a given affinity in μmol/g [36]. In the *N*(*K*)–log *K* plot (Figure 3b), both the MIP films exhibited a uniform decaying form, indicating the binding affinity features of heterogeneous surfaces commonly found in the exponentially tailing portion of the binding isotherm with a broad unimodal distribution.

### 3.4. Selectivity

**Figure 4:**
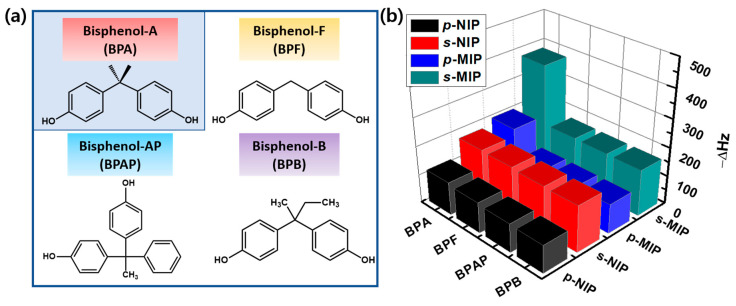
Comparative selectivity of the QCM sensors: (**a**) chemical structures of BPA, BPF, BPAP, and BPB and (**b**) the QCM sensing response (−∆*f*) of BPA, BPF, BPAP, and BPB solutions (225 nM) is shown for a 1-h measurement period on BPA-imprinted sensors (*p*-MIP and *s*-MIP films) and non-imprinted sensors (*p*-NIP and *s*-NIP films).

The specific selectivity of the two BPA-imprinted MIP films was investigated by comparing the sensing response of similar chemical compounds (Figure 4a). The ∆*f* values of the MIP and NIP films for a 1-h rebinding process were explored in 225-nM aqueous solutions with chemically similar interfering agents (BPF, BPAP, or BPB) (Figures S9 and S10). As shown in Figure 4b, the ∆*f* values for the sensing response of the BPA molecules increased for the MIP films, whereas the ∆*f* values in the MIP films were almost constant for the three other chemicals: 104 Hz for the *p*-MIP film and 164 Hz for the *s*-MIP film. In this sensing process, nitrogen in pyridine can interact with hydroxyl groups in all bisphenol derivatives with similar structures through hydrogen bonding. However, as 4-VP interacts with BPA, van der Waals force and π–π interaction also contribute to the selective recognition of BPA in the cavities of the MIP films. Similarly, the ∆*f* values of the NIP films were almost identical for the sensing responses of all the chemicals because of the major influence of non-specific binding; additionally, the ∆*f* values attributed to the binding feature depended on the surface areas of the two NIP films. The selectivity coefficients (k*) (i.e., ∆*f*_BPA_/∆*f*_(BPB, BPAP, or BPF)_) of the two MIP films increased in the following order: 1.897 (BPF) < 1.892 (BPAP) < 1.912 (BPB) for the *p*-MIP film and 2.267 (BPF) < 2.293 (BPAP) < 2.300 (BPB) for the *s*-MIP film. Thus, the *s*-MIP film-based QCM sensor showed more enhanced selective recognition, which resulted in remarkable signal amplification.

## 4. Conclusions

Two BPA-imprinted poly(4-VP-co-EGDMA) (i.e., *s*-MIP and *p*-MIP films) were successfully fabricated on silica skeleton surfaces and gold pinholes of silica inverse opal using the SI-ATRP synthesis method. The sensing features (i.e., sensitivity and selectivity) of the two MIP films on large surface areas were investigated by measuring the ∆*f* values of the QCM sensors. The ∆*f* values for the *p*-MIP and *s*-MIP films were amplified (−199 ± 4.9 and −376 ± 19.1 Hz, respectively), whereas for *p*-/*s*-NIP films, the ∆*f* values were obtained in a relatively lower range, between −115 ± 19.2 and −174 ± 5.8 Hz, by the influence of non-specific adsorption on the surface of the films. In addition, the IF for the *p*-MIP and *s*-MIP films was 1.72 and 2.15, respectively. Based on the ∆*f* value obtained, the LOQ and LOD values were calculated to be 54.924 and 18.125 nM (*p*-MIP film) and 38.419 and 12.678 nM (*s*-MIP film), respectively. Finally, the selectivity coefficients (k*) were calculated to be in the range 1.897–1.912 for the *p*-MIP film and 2.267–2.300 for the *s*-MIP film. From all the results, with respect to signal amplification of the QCM sensors, the *s*-MIP film showed better sensing features and faster detection response than the *p*-MIP film. Moreover, the Q_e_ value of the *p*-MIP film was greater than that of the *s*-MIP film because of the difference in the total MIP mass grown from different active substrate areas.

## Supplementary Materials

The following are available online at https://www.mdpi.com/2073-4360/12/9/1892/s1, Figure S1: SEM image of silica inverse opal; Figure S2: AFM images of (a) s-MIP film on the only silica surface fabricated by sol-gel processing and (b) p-MIP film grown from the gold pin-holes of silica inverse opal; Figure S3: Adsorbed BPA mass in mg per poly(4-VP-co-EGDMA) unit weight in g (Qe) as a function of time on the surface of the two MIP films (namely, s-MIP and p-MIP films) in a 225 nM BPA aqueous solution during equilibrium for a 1 h rebinding process (n = 3); Figure S4: Frequency change (∆f) as a function of time on the surfaces of two NIP films (namely, s-NIP and p-NIP films) in a 225 nM BPA aqueous solution for a 1 h rebinding process (n = 3); Figure S5: Frequency change (∆f) as a function of time on the surfaces of two MIP films ((a) s-MIP and (b) p-MIP films) in various concentrations of BPA (45–225 nM) for a 1 h rebinding process (n = 3); Figure S6: Binding capacity (mg/g) as a function of time on the surfaces of two MIP films (s-MIP and p-MIP films) for various concentrations of BPA (45–225 nM) for a 1 h rebinding process (n = 3); Figure S7: Frequency change (∆f) as a function of time on the surfaces of (a) s-NIP and (b) p-NIP films for various concentrations of BPA (45–225 nM) for a 1 h rebinding process (n = 3); Figure S8: Frequency change (−∆f) on the surfaces of s-NIP and p-NIP films as a function of the initial BPA concentration (C0, 45–225 nM) (n = 3); Figure S9: Frequency change (∆f) as a function of time on the surfaces of (a) s-MIP and (b) p-MIP films for BPA, BPF, BPAP, and BPB solutions at a fixed concentration (225 nM) for a 1 h rebinding process (n = 3); Figure S10: Frequency change (∆f) as a function of time on the surfaces of (a) s-NIP and (b) p-NIP films for BPA, BPF, BPAP, and BPB solutions at a fixed concentration (225 nM) for a 1 h rebinding process (n = 3).
