# Supplementary material for: Molecular Imprinting of Bisphenol A on Silica Skeleton and Gold Pinhole Surfaces in 2D Colloidal Inverse Opal through Thermal Graft Copolymerization
Source: Polymers (Basel). 2020 Aug 22;12(9):1892. doi: 10.3390/polym12091892 (PMC7564607; doi:10.3390/polym12091892)
Supplement: Supplementary file 1 [file polymers-12-01892-s001.pdf]

# Supplementary Materials

## Molecular Imprinting of Bisphenol A on Silica Skeleton and Gold Pinholes Surfaces in 2D Colloidal Inverse Opal through Thermal Graft Copolymerization

**Jin Chul Yang[a] and Jinyoung Park[a,b]***

[a]*School of Applied Chemical Engineering, Kyungpook National University, 80 Daehak-ro, Buk-gu, Daegu 41566, Republic of Korea*

[b]*Department of Polymer Science & Engineering, Kyungpook National University, 80 Daehak-ro, Buk-gu, Daegu 41566, Republic of Korea*

*Corresponding Author

Tel.: +82 53 950 5624; Fax: +82 53 950 6623

*E-mail address*: jinpark@knu.ac.kr (J. Park)

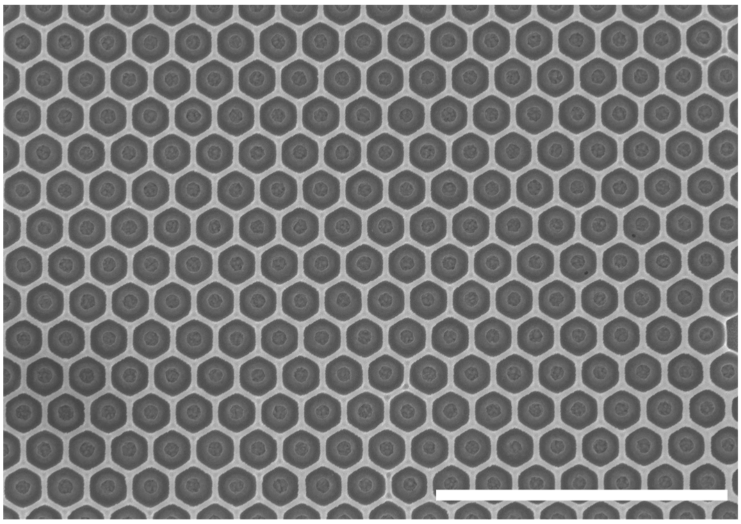

**Figure S1**. SEM image of silica inverse opal. The scale bar is 5μm.

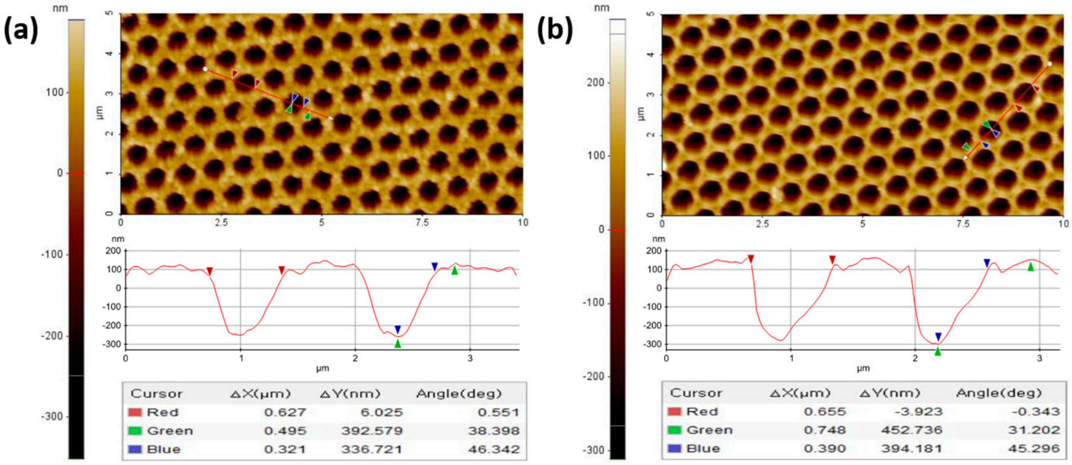


**Figure S2**. AFM images of (a) *s*-MIP film on the only silica surface fabricated by sol-gel processing and (b) *p*-MIP film grown from the gold pin-holes of silica inverse opal. AFM line profilometry is included in each AFM image.

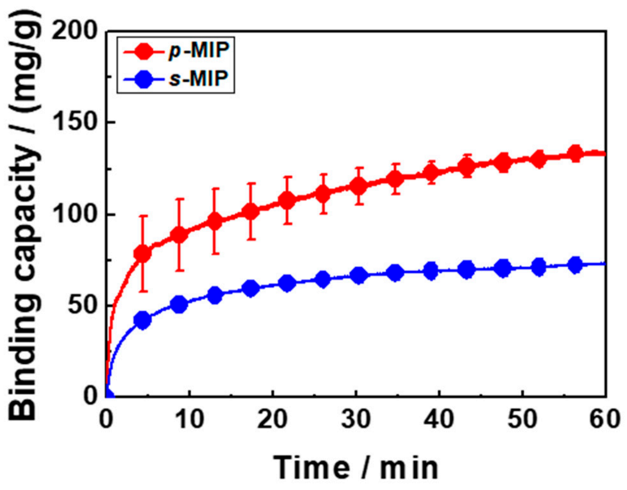


**Figure S3**. Adsorbed BPA mass in mg per poly(4-VP-co-EGDMA) unit weight in g ($Q_e$) as a function of time on the surface of the two MIP films (namely, *s*-MIP and *p*-MIP films) in a 225-nM BPA aqueous solution during equilibrium for a 1-h rebinding process (n =3).

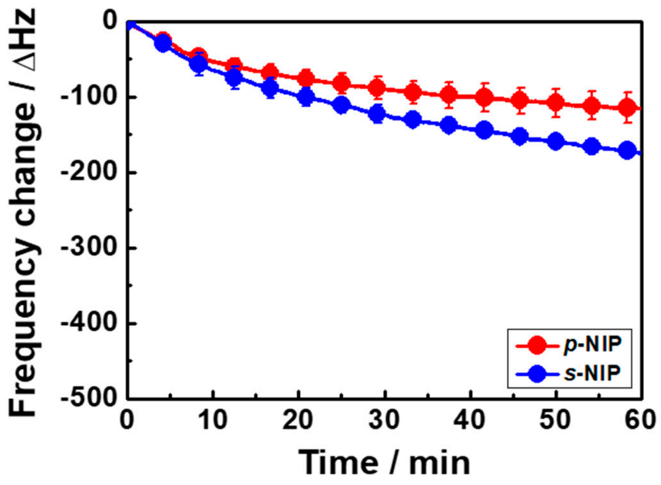


**Figure S4**. Frequency change ($\Delta f$) as a function of time on the surfaces of two NIP films (namely, *s*-NIP and *p*-NIP films) in a 225-nM BPA aqueous solution for a 1-h rebinding process (n = 3).

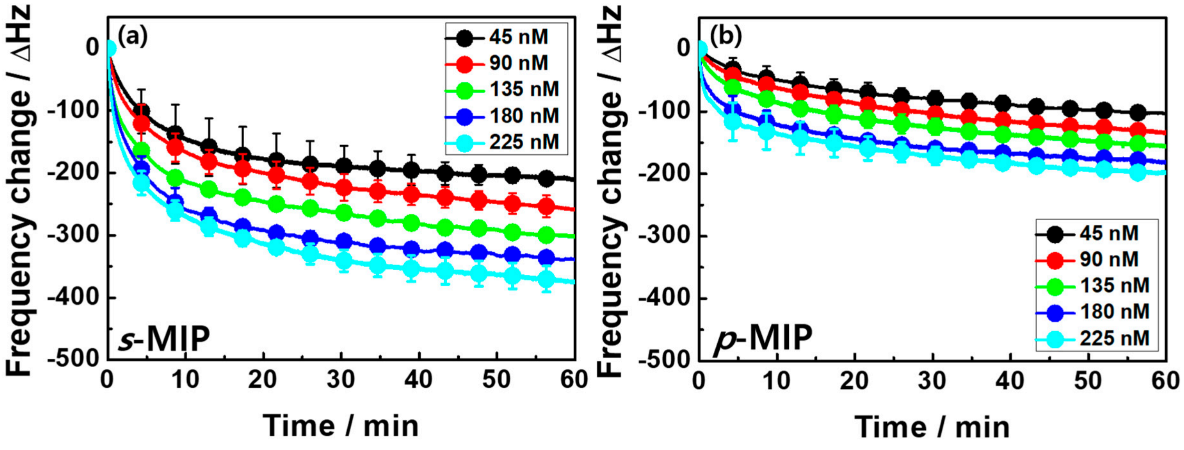


**Figure S5**. Frequency change ($\Delta f$) as a function of time on the surfaces of two MIP films ((a) *s*-MIP and (b) *p*-MIP films) in various concentrations of BPA (45–225 nM) for a 1-h rebinding process (n = 3).

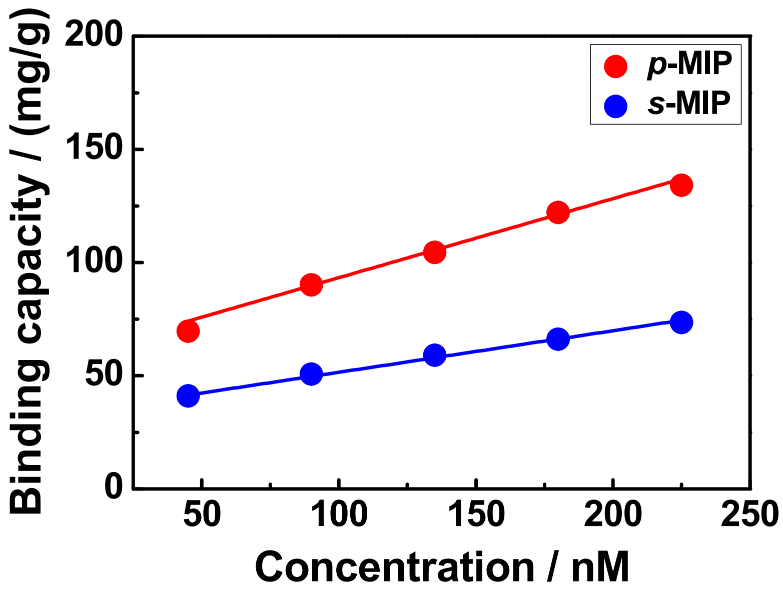


**Figure S6**. Binding capacity (mg/g) as a function of time on the surfaces of two MIP films (*s*-MIP and *p*-MIP films) for various concentrations of BPA (45–225 nM) for a 1-h rebinding process (n = 3).

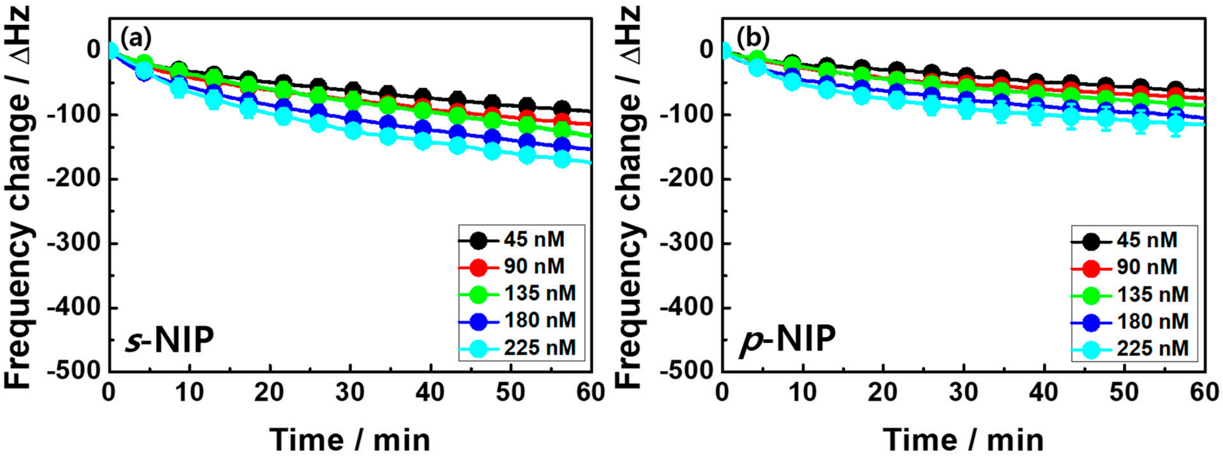


**Figure S7**. Frequency change (Δ*f*) as a function of time on the surfaces of (a) *s*-NIP and (b) *p*-NIP films for various concentrations of BPA (45 – 225 nM) for a 1-h rebinding process (n = 3).

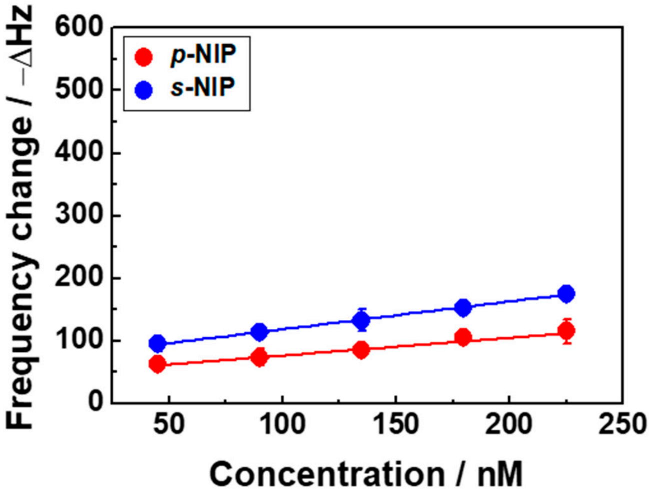


**Figure S8**. Frequency change (−Δ*f*) on the surfaces of *s*-NIP and *p*-NIP films as a function of the initial BPA concentration ($C_0$, 45 – 225 nM) (n = 3).

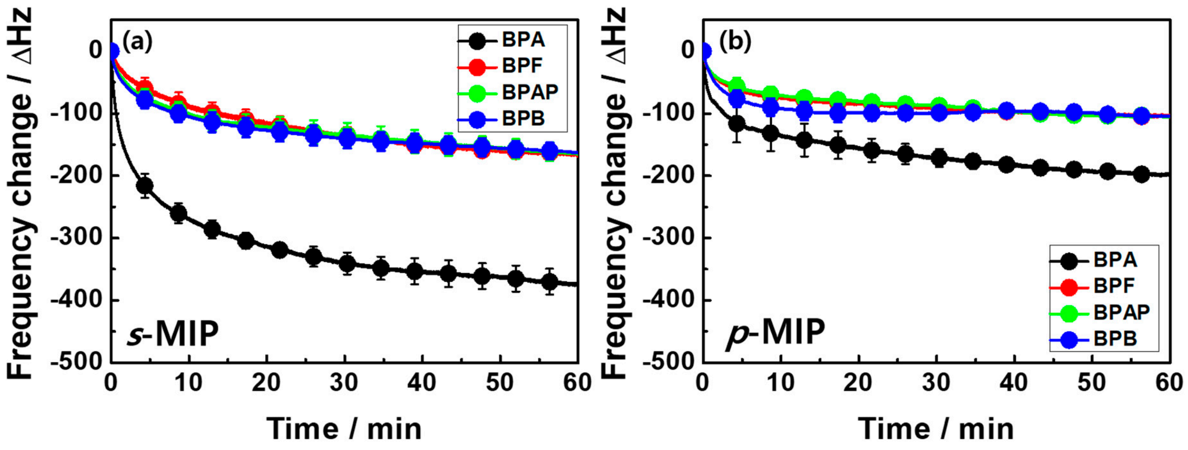


**Figure S9**. Frequency change ($\Delta f$) as a function of time on the surfaces of (a) *s*-MIP and (b) *p*-MIP films for BPA, BPF, BPAP, and BPB solutions at a fixed concentration (225 nM) for a 1-h rebinding process (n = 3).

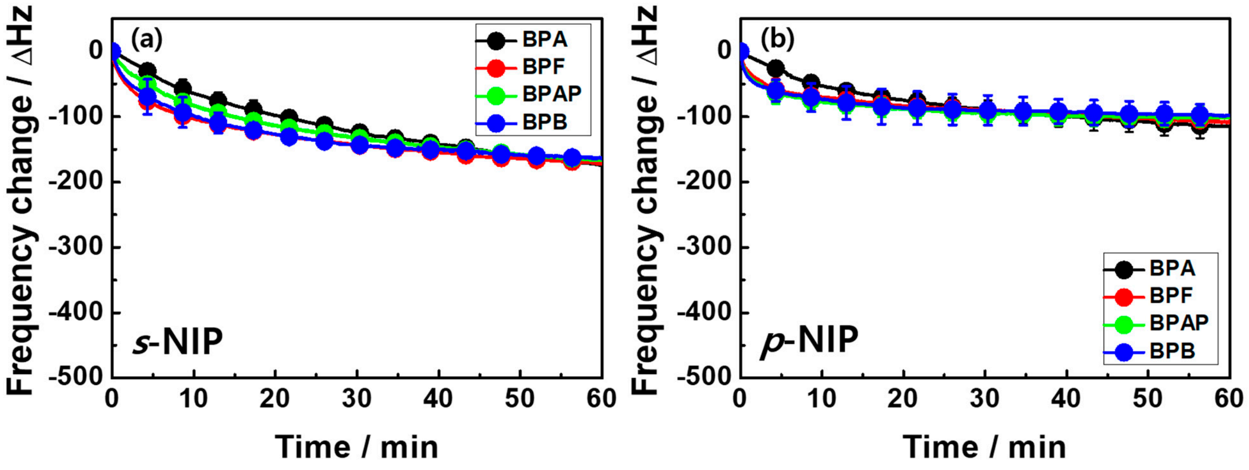


**Figure S10**. Frequency change ($\Delta f$) as a function of time on the surfaces of (a) *s*-NIP and (b) *p*-NIP films for BPA, BPF, BPAP, and BPB solutions at a fixed concentration (225 nM) for a 1-h rebinding process (n = 3).
